# Recombinant Macrophage Migration Inhibitory Factor Derived from *Trichinella spiralis* Suppresses Obesity by Reducing Body Fat and Inflammation

**DOI:** 10.3390/ijms27020887

**Published:** 2026-01-15

**Authors:** Seo Yeong Choi, Mi-Kyung Park, Yu Jin Jeong, Dong Gyu Han, Chaeeun Jin, Chang Woo Han, Se Bok Jang, Shin Ae Kang, Hak Sun Yu

**Affiliations:** 1 Department of Parasitology and Tropical Medicine, School of Medicine, Pusan National University, Yangsan 50612, Republic of Korea; tjdud2080@naver.com (S.Y.C.); alrud9697@pusan.ac.kr (M.-K.P.); ujin1139@naver.com (Y.J.J.); gyuya1021@naver.com (D.G.H.); cjin2523@pusan.ac.kr (C.J.); 2 Department of Molecular Biology, College of Natural Sciences, Pusan National University, Busan 46241, Republic of Korea; hotorses@pusan.ac.kr (C.W.H.); sbjang@pusan.ac.kr (S.B.J.); 3 Institute of Systems Biology, Pusan National University, Busan 46241, Republic of Korea; 4 Department of Environmental Medical Biology, Catholic Kwandong University, Gangneung 25601, Republic of Korea; sakang@cku.ac.kr; 5 Research Institute for Convergence of Biomedical Science and Technology, Pusan National University Yangsan Hospital, Yangsan 50612, Republic of Korea

**Keywords:** *Trichinella spiralis*, macrophage migration inhibitory factor, rTs-MIF, obesity, inflammation

## Abstract

Obesity, an escalating global health crisis, is characterized by adipose tissue hypertrophy and chronic low-grade inflammation. Although anti-obesity drugs can induce weight loss, their use is limited by adverse effects, underscoring the need for safer therapeutic strategies. In this study, we generated a recombinant form of *Trichinella spiralis*-derived macrophage migration inhibitory factor (rTs-MIF) and investigated its anti-inflammatory and anti-obesity effects via immunometabolic regulation. Male C57BL/6 mice fed a 45% high-fat diet were orally administered rTs-MIF, and its effects were evaluated by measuring fat mass, glucose metabolism, serum cytokines, liver histology, and adipose tissue parameters. In 3T3-L1 cells, we examined the effects of rTs-MIF on adipocyte differentiation, obesity-related gene expression, and intracellular signaling pathways. Oral rTs-MIF suppressed body weight gain, reduced fat mass, improved glucose levels, and decreased the food efficiency ratio. It also lowered pro-inflammatory cytokines and increased markers associated with M2 macrophages. In 3T3-L1 cells, rTs-MIF inhibited adipocyte differentiation and reduced the expression of lipogenic transcription factors and mouse *Mif* while modulating AKT and p44/42 MAPK signaling. These findings identify rTs-MIF as a potential bioactive candidate that ameliorates obesity by regulating the immune–metabolic axis.

## 1. Introduction

Obesity is a major risk factor for chronic diseases, including cardiovascular diseases, diabetes, and hypertension [1,2,3]. Since the 1990s, obesity occurrences have more than doubled in children and tripled in adolescents [4]. If this trend continues, it is estimated that 51% of the global population will be obese by 2030 [5]. This trend is evident in both developed and developing regions, and the demand for anti-obesity therapies continues to rise [6,7]. Glucagon-like peptide-1 (GLP-1)-based drugs have recently attracted significant attention as anti-obesity therapeutics [8]; however, their long-term use is associated with pancreatitis, thyroid cancer, and gastrointestinal complications [9,10].

Obesity is not merely a state of energy imbalance, but is also characterized by chronic low-grade inflammation within the adipose tissue [11]. Classically activated M1 macrophages produce pro-inflammatory cytokines such as IL-6, IL-1β, and TNF-α; meanwhile, activated M2 macrophages secrete IL-10 and TGF-β, thereby promoting tissue repair and anti-inflammatory responses [12]. Adipocytes themselves also produce pro-inflammatory cytokines [13,14]. Interestingly, various helminth infections are known to induce Th2 or Treg-biased immune responses, suppress inflammation, and restore tissue homeostasis [15,16]. Previous studies have shown that *Trichinella spiralis* infection suppressed body weight gain and alleviated adipose tissue inflammation in high-fat diet (HFD)-induced obese mouse models [17]. Moreover, the total lysate of *T. spiralis* has been reported to reduce body weight and fat mass and improve blood glucose levels [18]. Accordingly, we focused on helminth-derived macrophage migration inhibitory factor (MIF) with immunoregulatory properties.

Host MIF is a well-known pro-inflammatory factor that binds to CD74 and activates inflammatory signaling pathways [19]. At the molecular level, MIF activates the ERK1/2 MAPK pathway via CD74/CD44 [20], inducing the production of pro-inflammatory cytokines such as TNF-α, IL-1β, IL-6, and IFN-γ and amplifying immune responses [21]. In contrast, helminth-derived MIFs have been reported to exert anti-inflammatory effects, including the suppression of allergic inflammation, attenuation of colitis, and modulation of immune homeostasis [22,23,24]. The structural features unique to the *T. spiralis* MIF (rTs-MIF) suggest functional properties distinct from those of the host MIF, highlighting its potential as a therapeutic target.

Moreover, MIF family proteins are small and characterized by relatively stable molecules with a trimer structure that can be easily mass produced in their recombinant form, making them attractive candidates for biological therapeutics [25]. As rTs-MIF does not directly target the pathways associated with adverse gastrointestinal, neurological, or cardiovascular effects commonly observed with current anti-obesity drugs, it is expected to have a lower risk of long-term side effects [26]. Meanwhile, parasite-derived immunomodulatory proteins have evolutionarily adapted to minimize host tissue damage, suggesting their high degree of safety and substantial potential for use as therapeutic agents [27].

Here, we investigated the potential of *T. spiralis*-derived MIF as a modulator of obesity and evaluated its feasibility as a novel anti-obesity therapeutic candidate with fewer adverse effects by targeting the immune–metabolic axis.

## 2. Results

### 2.1. Structural Differences Between T. spiralis MIF and M. musculus MIF

**Figure 1:**
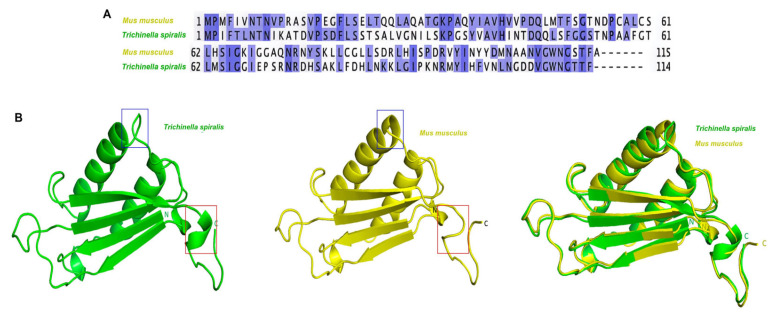
Sequence and structural comparison of the MIF associated with *T. spiralis* and *M. musculus*. (**A**) Amino acid sequence alignment of *T. spiralis* MIF and *M. musculus* MIF. Conserved residues between the two proteins are highlighted in blue, and the overall sequence identity is approximately 40%. (**B**) Predicted 3D structures generated using SWISS-MODEL (https://swissmodel.expasy.org/, 7 September 2023) and AlphaFold (https://alphafold.ebi.ac.uk/, 7 September 2023) were superimposed. Although the central β-sheet core is similar between the two proteins, the surface-exposed loop regions (red and blue boxes) differ. These structural differences may influence receptor interaction and immunoregulatory activity.

To analyze the similarity of MIF among these species, we performed multiple sequence alignments of *T. spiralis* and *M. musculus* (Figure 1A). The sequence identity between *T. spiralis* MIF and *M. musculus* MIF was 40.35%. A superimposition of *T. spiralis* MIF (green) and *M. musculus* MIF (yellow) is shown in the Figure (Figure 1B). The root-mean-square deviation (RMSD) between the two structures was 0.647 Å.

### 2.2. rTs-MIF Reduces Obesity in Mice

**Figure 2:**
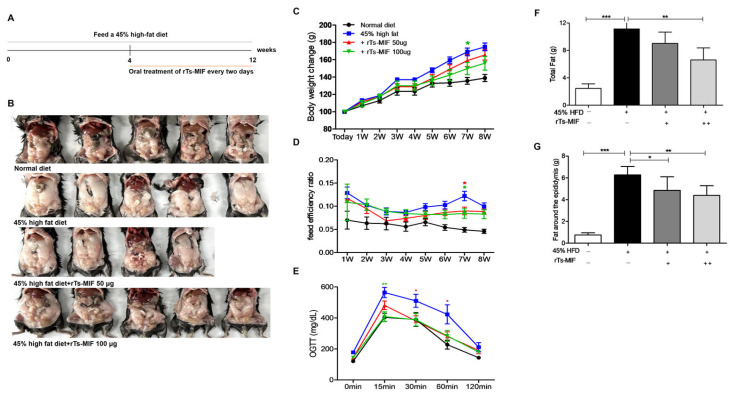
Anti-obesity effects of rTs-MIF in a 45% high-fat diet (HFD)-induced obesity model. (**A**) Experimental scheme. Mice were fed 45% HFD for 12 weeks. From week 4, rTs-MIF (50 or 100 µg) was administered orally every other day for 8 weeks. (**B**) Representative images of mice and abdominal adiposity in each group. HFD mice exhibited markedly increased abdominal fat, which was reduced in rTs-MIF-treated groups. (**C**) Changes in body weight over 8 weeks. rTs-MIF treatment attenuated HFD-induced weight gain compared with HFD controls. (**D**) Food efficiency ratio (FER = daily body weight gain (g)/daily food intake (g)), which was significantly lowered by rTs-MIF, particularly in week 7. (**E**) Oral glucose tolerance test results. rTs-MIF improved glucose tolerance in HFD-fed mice. (**F**) Total fat mass measured using a body composition analyzer. (**G**) Comparison of eWAT weight. ‘+’ indicates mice treated with 50 µg rTs-MIF, and ‘++’ indicates mice treated with 100 µg rTs-MIF. (n = 5 per group; * *p* < 0.05, ** *p* < 0.01, and *** *p* < 0.001).

The overall experimental scheme is presented in Figure 2A. After 8 weeks of rTs-MIF administration, body fat accumulation was reduced compared with that in the HFD group (Figure 2B). In the 100 µg group, body weight gain was significantly suppressed from week 7 onward (Figure 2C), and FER significantly decreased (Figure 2D). OGTT results showed improved glucose tolerance (Figure 2E). Consistently, the total fat mass measured by MRI and the eWAT weight were reduced in a dose-dependent manner in rTs-MIF-treated mice (Figure 2F,G).

### 2.3. rTs-MIF Inhibits Hepatic Lipid Accumulation and Lipogenic Gene Expression

**Figure 3:**
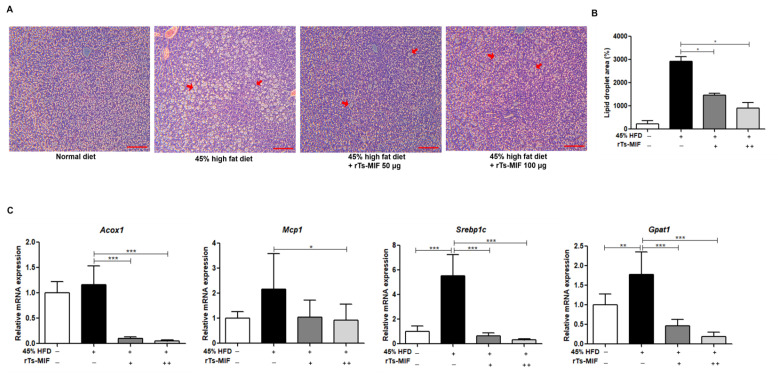
Inhibitory effect of rTs-MIF on hepatic lipid accumulation in a 45% HFD-induced obesity model. (**A**) Representative H&E-stained liver sections (100×, Scale bar = 50 µm). Large and numerous lipid droplets (red arrows) were observed in HFD mice, whereas lipid accumulation was markedly reduced in rTs-MIF-treated groups. (**B**) Quantification of hepatic lipid droplet area (%). (**C**) Hepatic expression of lipid metabolism-related genes (*Acox1*, *Mcp1*, *Srebp1c*, and *Gpat1*). HFD-induced upregulation of these genes was significantly attenuated by rTs-MIF treatment. ‘+’ indicates mice treated with 50 µg rTs-MIF, and ‘++’ indicates mice treated with 100 µg rTs-MIF. (n = 5 per group; * *p* < 0.05, ** *p* < 0.01, and *** *p* < 0.001).

H&E staining of liver sections revealed minimal lipid droplet formation in ND mice, whereas HFD mice exhibited a marked increase in intrahepatic lipid droplets. By contrast, mice treated with rTs-MIF (50 or 100 µg) showed a substantial reduction in the size and number of hepatic lipid droplets (Figure 3A). Quantitative analysis of the lipid droplet area confirmed that the pronounced HFD-induced increase in hepatic lipid accumulation was significantly attenuated by rTs-MIF treatment (Figure 3B). Gene expression analysis revealed that rTs-MIF downregulated HFD-induced expression of *Acox1*, *Mcp1*, *Srebp1c*, and *Gpat1* (Figure 3C). These findings indicate that rTs-MIF effectively suppressed hepatic lipogenesis and ameliorated HFD-induced metabolic disturbances in the liver.

### 2.4. rTs-MIF Decreases Th1 Cytokines and Increases Th2 Cytokines

**Figure 4:**
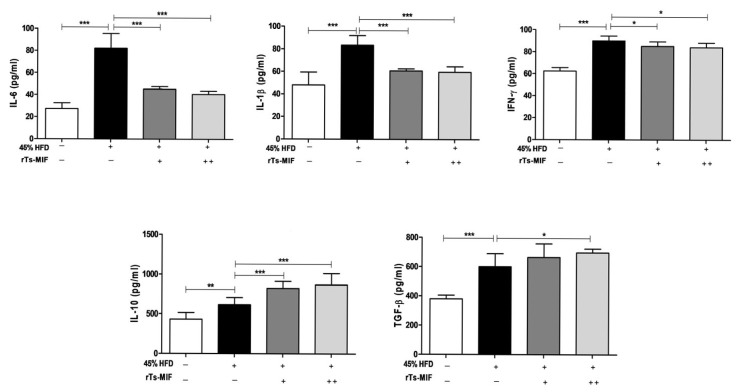
Effects of rTs-MIF on serum pro- and anti-inflammatory cytokine production. Serum cytokine concentrations were measured using ELISA. Pro-inflammatory cytokines (IL-6, IL-1β, and IFN-γ) were reduced in rTs-MIF-treated groups compared with HFD controls. Anti-inflammatory cytokines (IL-10 and TGF-β) were further increased in rTs-MIF-treated mice, indicating a shift toward an anti-inflammatory immune environment. ‘+’ indicates mice treated with 50 µg rTs-MIF, and ‘++’ indicates mice treated with 100 µg rTs-MIF. (n = 5 per group; * *p* < 0.05, ** *p* < 0.01, and *** *p* < 0.001).

The 45% HFD significantly increased serum levels of the pro-inflammatory cytokines IL-6, IL-1β, and IFN-γ. In contrast, treatment with rTs-MIF (50 or 100 µg) reduced the levels of these pro-inflammatory cytokines. Anti-inflammatory cytokines IL-10 and TGF-β were elevated in the HFD group and further increased in the rTs-MIF-treated groups, suggesting that rTs-MIF enhances anti-inflammatory responses and contributes to the resolution of HFD-induced inflammation (Figure 4).

### 2.5. rTs-MIF Modulates Macrophage Composition in White Adipose Tissue

**Figure 5:**
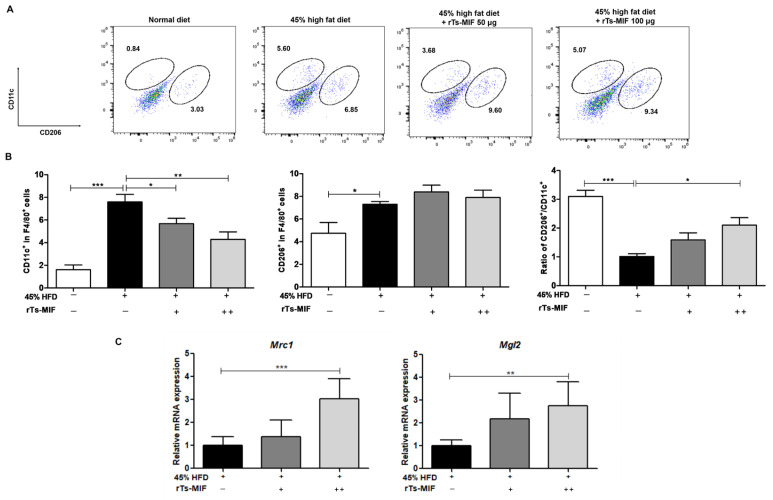
Regulatory effects of rTs-MIF on macrophage populations in adipose tissue. (**A**) Flow cytometric analysis of CD11c^+^ M1 and CD206^+^ M2 macrophages in the stromal vascular fraction (SVF) of epididymal white adipose tissue (eWAT). (**B**) Quantification of CD11c^+^F4/80^+^ (M1) and CD206^+^F4/80^+^ (M2) macrophages and calculation of the M2/M1 ratio. rTs-MIF treatment significantly reduced M1 macrophages and increased the M2/M1 ratio. (**C**) mRNA expression of M2-associated marker genes *Mrc1*, and *Mgl2* in eWAT, both of which were significantly upregulated in rTs-MIF-treated groups. ‘+’ indicates mice treated with 50 µg rTs-MIF, and ‘++’ indicates mice treated with 100 µg rTs-MIF. (n = 5 per group; * *p* < 0.05, ** *p* < 0.01, and *** *p* < 0.001).

Flow cytometric analysis revealed that high-fat diet (HFD) feeding markedly increased the proportion of CD11c^+^ macrophages within the stromal vascular fraction (SVF) of epididymal white adipose tissue (eWAT), indicating an accumulation of pro-inflammatory M1 macrophages. In contrast, mice treated with rTs-MIF at doses of 50 and 100 µg exhibited a reduction in CD11c^+^ cells together with an increase in CD206^+^ cells, suggesting a shift in macrophage composition toward an anti-inflammatory phenotype (Figure 5A). Quantitative assessment confirmed that rTs-MIF treatment significantly reduced the CD11c^+^F4/80^+^ M1 macrophage population, whereas CD206^+^F4/80^+^ M2 macrophages showed an increasing trend that did not reach statistical significance. Accordingly, the M2/M1 macrophage ratio was elevated in rTs-MIF-treated groups compared with HFD controls, indicating a relative enrichment of M2-like macrophages in adipose tissue (Figure 5B). Consistent with these cellular changes, mRNA expression levels of M2-associated markers, including *Mrc1 (encoding CD206)* and *Mgl2*, were significantly upregulated in the adipose tissue of rTs-MIF-treated mice. These molecular findings support the notion that rTs-MIF is associated with a shift in adipose tissue macrophage balance toward an anti-inflammatory M2 profile under HFD conditions (Figure 5C).

### 2.6. rTs-MIF Inhibits Adipocyte Differentiation in 3T3-L1 Cells

**Figure 6:**
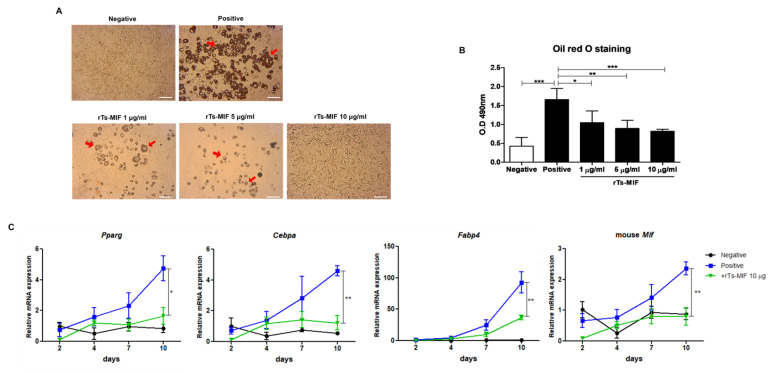
Inhibitory effects of rTs-MIF on 3T3-L1 adipocyte differentiation. (**A**) Oil Red O staining at day 10 of differentiation. Lipid droplets were markedly accumulated in the positive control group, whereas rTs-MIF reduced lipid accumulation in a dose-dependent manner. Red arrows indicate intracellular lipid droplets. Scale bar = 50 µm. (**B**) Quantification of Oil Red O staining (absorbance at 490 nm). (**C**) Expression of adipogenic marker genes (*Pparg*, *Cebpa*, and *Fabp4*) at days 2, 4, 7, and 10 of differentiation. The differentiation-induced positive control group exhibited increased expression of these genes, which was suppressed by rTs-MIF (10 µg/mL). (n = 4 wells per group, and three independent experiments were performed; * *p* < 0.05, ** *p* < 0.01, and *** *p* < 0.001).

Oil Red O staining demonstrated abundant lipid droplet formation in fully differentiated adipocytes in the positive control group, whereas rTs-MIF treatment (1, 5, and 10 µg/mL) reduced the size and number of lipid droplets in a dose-dependent manner (Figure 6A). Quantification of Oil Red O absorbance demonstrated that intracellular lipid accumulation was significantly decreased by rTs-MIF treatment (Figure 6B). During adipocyte differentiation, expression of the adipogenic transcription factors *Pparg, Cebpa, Fabp4,* and mouse *Mif* progressively increased in the positive control group, whereas this increase was attenuated in cells treated with 10 µg/mL rTs-MIF. The difference in the expression levels between the positive control and rTs-MIF groups was particularly pronounced at the late differentiation stage (day 10), demonstrating that rTs-MIF inhibited adipocyte differentiation (Figure 6C).

### 2.7. rTs-MIF Modulates Obesity-Related Signaling Pathways During Early 3T3-L1 Differentiation

**Figure 7:**
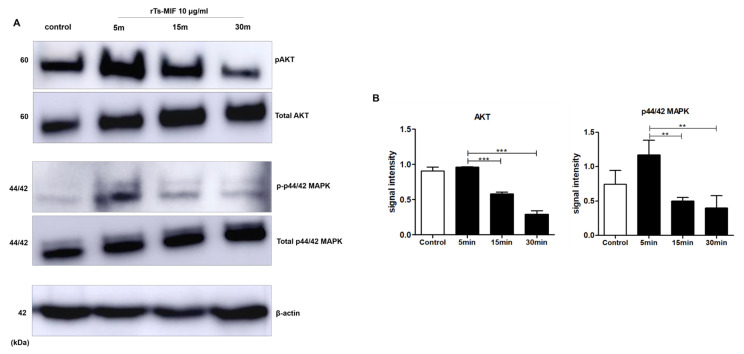
Modulatory effects of rTs-MIF on obesity-related signaling pathways. (**A**) Western blot analysis of AKT and p44/42 MAPK phosphorylation in 3T3-L1 cells treated with 10 µg/mL rTs-MIF for 5, 15, or 30 min. β-actin was used as a loading control. (**B**) Quantification of band intensities. Phosphorylation of both AKT and p44/42 MAPK increased transiently at 5 min and declined at later time points. (n = 3 independent experiments; ** *p* < 0.01, and *** *p* < 0.001).

Time-course analysis of signaling pathways after rTs-MIF (10 µg/mL) treatment revealed that phosphorylation of AKT and p44/42 MAPK tended to increase at 5 min but decreased at 15–30 min. The total AKT and total p44/42 MAPK levels remained relatively unchanged over time (Figure 7A). Quantification of band intensities confirmed a transient increase in phosphorylation of AKT and p44/42 MAPK at early time points, followed by a decline at later time points (Figure 7B).

## 3. Discussion

In this study, we comprehensively evaluated the anti-obesity and anti-inflammatory effects of rTs-MIF at systemic, tissue, and cellular levels. We demonstrated that rTs-MIF suppressed body weight gain, reduced fat accumulation, alleviated hepatic lipid deposition, decreased systemic inflammation, promoted macrophage polarization toward M2, inhibited adipocyte differentiation, downregulated lipogenic transcription factors, and modulated AKT and p44/42 MAPK signaling in HFD-induced obese mice and 3T3-L1 cells.

HFD-fed mice exhibited continuous body weight gain compared to ND controls, whereas rTs-MIF-treated mice showed attenuated weight gain. The FER analysis indicated reduced weight gain relative to food intake. Although the food efficiency ratio (FER) can be influenced by factors such as nutrient absorption and intestinal microflora, the observed changes were interpreted in the context of longitudinal analyses of body weight and food intake using repeated-measures statistical approaches. Improvement in glucose tolerance was consistent with previous observations that helminth-derived molecules support metabolic homeostasis [28,29]. These effects extend beyond simple weight reduction and align with an anti-obesity pattern wherein reduced metabolic stress and inflammation contribute to improved insulin sensitivity [30,31,32]. Liver histology showed that rTs-MIF markedly reduced HFD-induced hepatic lipid droplet accumulation, suggesting attenuation of fatty liver and improvement of hepatic lipid metabolism through the suppression of lipogenesis and/or enhancement of fatty acid oxidation. HFD typically upregulates lipogenesis-related genes such as *Acox1*, *Mcp1*, *Srebp1c*, and *Gpat1* in the liver, promoting lipid accumulation [33,34,35,36]. Although reductions in hepatic lipid droplet area were accompanied by coordinated changes in lipid metabolism-related gene expression, correlation analyses between gene expression levels and lipid droplet area may help clarify the underlying mechanisms in future studies. In this study, rTs-MIF reversed these gene expression changes, thus mitigating HFD-induced hepatic metabolic stress and facilitating the restoration of lipid homeostasis. In rTs-MIF-treated mice, reductions in IL-6, IL-1β, and IFN-γ, along with further increases in IL-10 and TGF-β, clearly showed a shift from a pro-inflammatory to an anti-inflammatory systemic cytokine profile. Obesity is generally associated with elevated IL-6, IL-1β, and IFN-γ levels due to an increased M1 macrophage population and chronic low-grade inflammation [37]. Meanwhile, IL-10 and TGF-β may increase as part of an adaptive immunoregulatory response aimed at limiting tissue damage and stress under chronic inflammatory conditions [38]. The additional increase in anti-inflammatory cytokines in rTs-MIF-treated mice suggests that helminth-derived MIF directly enhances anti-inflammatory signaling, rather than merely reflecting passive compensation. FACS demonstrated that rTs-MIF reduced the HFD-induced accumulation of CD11c^+^ M1 macrophages in adipose tissue and promoted an increase in CD206^+^ M2 macrophages. The elevated M2/M1 ratio in rTs-MIF-treated mice indicated that rTs-MIF actively reprogrammed macrophage polarization toward an anti-inflammatory phenotype. The increased *Mrc1*, and *Mgl2* expression supports the notion that rTs-MIF promotes M2 polarization. Together, these findings suggest that rTs-MIF alleviates the inflammatory microenvironment in the adipose tissue, contributing to improved adipocyte function and systemic metabolic normalization.

In vitro, rTs-MIF reduced lipid accumulation in 3T3-L1 adipocytes and suppressed *Pparg*, *Cebpa*, and *Fabp4* expression. *Pparg*, *Cebpa*, and *Fabp4* are key adipogenic transcription factors and markers of adipocyte differentiation and maturation [39]. Furthermore, rTs-MIF treatment significantly suppressed the expression of mouse *Mif*, which typically increases during adipocyte differentiation. Because host MIF is known to promote adipogenesis and amplify inflammatory signaling [40], this finding provides important evidence that *T. spiralis* MIF may counter-regulate host MIF signaling, contributing to its anti-inflammatory and anti-obesity effects. Western blot analysis revealed that rTs-MIF induced a transient increase in AKT and p44/42 MAPK phosphorylation at 5 min, which decreased subsequently. The AKT and p44/42 MAPK signaling pathways are critical early regulators of adipocyte differentiation [41,42]. The observed transient changes in AKT and p44/42 MAPK phosphorylation suggest that rTs-MIF may interfere with the maintenance of sustained signaling required for adipogenesis, rather than inducing prolonged activation of these pathways. Host MIF is known to strongly activate p44/42 MAPK via CD74–CD44 and promote inflammation [43]. In contrast, rTs-MIF in this study induced only transient activation, which was followed by downregulation of these pathways. This suggests that helminth-derived MIF competitively interferes with host MIF or modulates receptor interactions in a manner that attenuates pro-inflammatory signaling, which in turn likely contributes to the inhibition of adipocyte differentiation, regulation of macrophage polarization, and mitigation of inflammation. These findings are in line with previous reports that helminth-derived MIFs exhibit distinct immunoregulatory and metabolic functions, despite their structural similarity to host MIF [43,44]. Differences in the exposed loop regions of helminth MIF may differentially affect its interactions with CD74 or other receptors, thereby conferring anti-inflammatory and anti-obesity properties.

Taken together, these findings suggest that rTs-MIF may act as a molecular regulator involved in both immune modulation and metabolic control, rather than functioning solely as an inhibitor of adipogenesis. Further studies focusing on receptor-level interactions, including CD74–CD44 engagement, as well as structural–functional analyses of helminth-derived MIF, will be crucial in defining the mechanisms underlying its immunometabolic effects more precisely. In addition, long-term and tissue-specific investigations may help clarify how rTs-MIF contributes to the maintenance of immune–metabolic homeostasis in obesity.

## 4. Materials and Methods

### 4.1. Protein Expression and Purification

Primers targeting the *T. spiralis* MIF gene (XM_003378364.1) were designed as follows: forward, 5′-GAATTCATGCCGATTTTTAC-3′; reverse, 5′-CTCGAGTCAGAATGTAGTACC-3′. The target gene was amplified using PCR, cloned into a T-vector, and subcloned into the pET-28a vector (Novagen, Darmstadt, Germany). The recombinant construct was transfected into *Escherichia coli* BL21 cells. Protein expression was induced with 0.5 mM IPTG (Sigma-Aldrich, St. Louis, MO, USA), and the cells were harvested via centrifugation (4000 rpm, 20 min, 4 °C). The cell pellets were disrupted using an ultrasonic homogenizer, and the supernatants were collected. The recombinant His-tagged protein was purified using a His-tag affinity column (Sigma-Aldrich, St. Louis, MO, USA), followed by endotoxin removal using an LPS depletion column (Thermo Fisher Scientific, Waltham, MA, USA).

### 4.2. Mice and Diet

Six-week-old, male C57BL/6 mice were purchased from Samtako (Seongnam-si, Republic of Korea). Mice were randomly allocated to groups and fed either a normal diet (ND) or a 45% high-fat diet (HFD; Research Diets, New Brunswick, NJ, USA) until the end of the experiment. After 4 weeks of HFD feeding, a subset of HFD-fed mice received oral rTs-MIF at doses of 50 or 100 µg every other day for 8 weeks. rTs-MIF was administered mixed with corn oil, with corn oil accounting for one-third of the total administration volume. Control HFD mice received an equal volume of vehicle consisting of PBS mixed with corn oil in the same proportion. The body weight and food intake were measured weekly. The food efficiency ratio (FER) was calculated as daily body weight gain (g)/daily food intake (g). All animal procedures were approved by the Institutional Animal Care and Use Committee (IACUC) of Pusan National University (Approval No. PNU-2025-0305).

### 4.3. Measurement of Body Fat Mass and Adipose Tissue Weight

One day before sacrifice, total body fat mass was quantified using a body composition analyzer (Bruker Minispec LF50, Billerica, MA, USA). After sacrifice, the epididymal white adipose tissue (eWAT) surrounding the testes was dissected and weighed to assess changes in fat mass.

### 4.4. Oral Glucose Tolerance Test (OGTT)

After 8 weeks of rTs-MIF administration, mice were fasted for 16 h and then orally gavaged with a 20% glucose solution at a dose of 10 µL per gram of body weight. Blood glucose levels were measured at 0, 15, 30, 60, and 120 min after glucose administration.

### 4.5. Histological Analysis

Liver samples were fixed in 4% formalin, embedded in paraffin, sectioned, and stained with hematoxylin and eosin (H&E) for histological evaluation. Lipid droplets in H&E-stained liver sections were quantified using ImageJ (version 1.54g; NIH, Bethesda, MD, USA). Images were captured at the same magnification, and the droplet area was measured as a percentage of the total hepatic tissue area. For each animal, three to five randomly selected fields were analyzed.

### 4.6. RNA Extraction and cDNA Synthesis

Total RNA was extracted from the liver tissue, eWAT, and 3T3-L1 cells using TRIzol reagent (Invitrogen, Burlington, ON, Canada). Quantitative PCR was performed using SYBR™ Select Master Mix (Thermo Fisher Scientific, Waltham, MA, USA). *Gapdh* was used as an endogenous control. The genes analyzed included *Acox1*, *Mcp1*, *Srebp1c*, *Gpat1*, *Pparg*, *Cebpa*, *Fabp4*, mouse *Mif*, *Mrc1*, and *Mgl2*. The primer sequences used for each gene are listed in Table 1.

### 4.7. Cytokine Analysis Using ELISA

At sacrifice, blood was collected from the heart, and the serum was separated by centrifugation. Serum concentrations of IL-6, IL-1β, IFN-γ, IL-10, and TGF-β were measured using ELISA kits (Thermo Fisher Scientific, Waltham, MA, USA) according to the manufacturer’s instructions.

### 4.8. Flow Cytometry (FACS)

eWAT was excised and enzymatically digested with 0.2% collagenase type I (Sigma-Aldrich, St. Louis, MO, USA) to isolate the stromal vascular fraction (SVF). Isolated SVF cells were stained with fluorophore-conjugated antibodies against F4/80, CD11c, and CD206 (eBioscience, San Diego, CA, USA). Macrophages were identified as F4/80^+^ cells, and CD11c and CD206 were used as representative markers of M1 and M2 macrophage phenotypes, respectively. Data acquisition was performed using a BD FACSCanto II flow cytometer (BD Biosciences, Franklin Lakes, NJ, USA), and data analysis was conducted using FlowJo software (version 10; BD Biosciences) according to the manufacturer’s instructions. CD11c and CD206 were selected as representative markers of classically activated (M1) and alternatively activated (M2) macrophages, respectively, as these markers are widely used to assess macrophage polarization in adipose tissue inflammation and metabolic disease models [11,12]. Detailed information regarding antibody specifications, gating strategy, and flow cytometry controls is provided in Supplementary Figure S1 and Supplementary Table S1.

### 4.9. 3T3-L1 Cell Culture and Differentiation

3T3-L1 preadipocytes were seeded in 24-well plates at a density of 5 × 10^4^ cells/well and cultured in DMEM supplemented with 10% FBS and penicillin/streptomycin until reaching 100% confluence. Upon confluence, adipocyte differentiation was induced for 48 h using DMEM containing 0.5 mM IBMX, 1 μM dexamethasone, and 1 μg/mL insulin with 10% FBS. rTs-MIF (1, 5, or 10 μg/mL) was co-treated throughout the differentiation period. After 48 h, cells were switched to maintenance medium (DMEM containing 1 μg/mL insulin), and the medium was replaced every 48 h until day 10. Lipid accumulation was assessed on day 10 by Oil Red O staining, and the expression of adipocyte differentiation-related genes was evaluated on days 2, 4, 7, and 10.

### 4.10. Oil Red O Staining

After 10 d of differentiation, the cells were washed with PBS and fixed with 4% formalin. The lipid droplets were stained with Oil Red O solution (0.5 g/L). The dye was then eluted with 100% isopropanol, and the absorbance was measured at 490 nm.

### 4.11. Western Blot Analysis

3T3-L1 cells were treated with 10 µg/mL rTs-MIF for 5, 15, or 30 min, and total protein was extracted. Proteins were separated using sodium dodecyl sulfate-polyacrylamide gel electrophoresis and transferred onto PVDF membranes. Membranes were probed with primary antibodies against phospho-AKT, total AKT, phospho-p44/42 MAPK, total p44/42 MAPK, and actin (Cell Signaling Technology, Danvers, MA, USA), followed by incubation with HRP-conjugated secondary antibodies. β-actin was used as a loading control for protein normalization. Signals were detected using WESTSAVE™ (AbFrontier, Seoul, Republic of Korea), and band intensities were quantified using ImageJ software.

### 4.12. Statistical Analysis

Data are presented as mean ± standard deviation (SD). For comparisons among three or more groups, one-way analysis of variance (ANOVA) followed by Dunnett’s multiple comparisons post hoc test was used to compare all treatment groups with the HFD control group. For repeated measurements over time, two-way repeated-measures ANOVA was applied. All statistical analyses were performed using GraphPad Prism 5 (GraphPad Software, San Diego, CA, USA). Statistical significance was set at *p* < 0.05.

## 5. Conclusions

In summary, rTs-MIF exerted anti-obesity effects by reducing body fat mass, suppressing hepatic steatosis, attenuating systemic and adipose tissue inflammation, inhibiting adipocyte differentiation, downregulating AKT and p44/42 MAPK signaling, and promoting an M2-dominant macrophage milieu in adipose tissue.

Given that current anti-obesity drugs are limited by adverse effects, high cost, and the need for long-term administration, rTs-MIF represents a promising new class of anti-obesity candidates that target the immune–metabolic axis. This study suggests that helminth-derived proteins may be broadly applicable as therapeutics for metabolic and immune-mediated diseases beyond obesity.

## 6. Patents

A patent application related to Ts-MIF and its potential therapeutic use has been filed. Seo Yeong Choi, Mi-Kyung Park, Yu Jin Jeong, Shin Ae Kang, and Hak Sun Yu are listed as inventors.

## Figures and Tables

**Table 1:** Primer sequences for quantitative PCR.

Primer	Sequence
*Gapdh*-for ^1^	5′-CAT CAC TGC CAC CCA GAA GAC TG-3′
*Gapdh*-rev	5′-ATG CCA GTG AGC TTC CCG TTC AG-3′
*Acox1*-for	5′-GCC ATT CGA TAC AGT GCT GTG AG-3′
*Acox1*-rev	5′-CCG AGA AAG TGG AAG GCA TAG G-3′
*Mcp1*-for	5′-GCT ACA AGA GGA TCA CCA GCA G-3′
*Mcp1*-rev	5′-GTC TGG ACC CAT TCC TTC TTG G-3′
*Srebp1c*-for	5′-GGA GCC ATG GAT TGC ACA TT-3′
*Srebp1c*-rev	5′-GGC CCG GGA AGT CAC TGT-3′
*Gpat1*-for	5′-GCA AGC ACT GTT ACC AGC GAT C-3′
*Gpat1*-rev	5′-TGC AAT CAG CCT TCG TCG GAA G-3′
*Mgl2*-for	5′-CGA GAC TTG AGC CAG AAG GTG A-3′
*Mgl2*-rev	5′-GCC TTC AAG TCT GTC TCC AGC T-3′
*Mrc1*-for	5′-CAG GTG TGG GCT CAG GTA GT-3′
*Mrc1*-rev	5′-TGT GGT GAG CTG AAA GGT GA-3′
*Pparg*-for	5′-GCC TCC TGG TGA CTT TAT GGA-3′
*Pparg*-rev	5′-GCA GCA GGT TGT CTT GGA TG-3′
*Cebpa*-for	5′-CAA AGC CAA GAA GTC GGT GGA CAA-3′
*Cebpa*-rev	5′-TCA TTG TGA CTG GTC AAC TCC AGC-3′
*Fabp4*-for	5′-TGA AAT CAC CGC AGA CGA CAG G-3′
*Fabp4*-rev	5′-GCT TGT CAC CAT CTC GTT TTC TC-3′
mouse *Mif*-for	5′-ATG CCT ATG TTC ATC GTG-3′
mouse *Mif*-rev	5′-TCA AGC GAA GGT GGA ACC-3′

^1^ for: forward; rev: reverse.

## Data Availability

The original contributions presented in this study are included in the article/Supplementary Materials. Further inquiries can be directed to the corresponding author.

## Supplementary Materials

The following supporting information can be downloaded at: https://www.mdpi.com/article/10.3390/ijms27020887/s1.

## References

[1] Felipe Parra Velasco P., Gaze D.C. (2023). Obesity and Cardiovascular Risk. Novel Pathogenesis and Treatments for Cardiovascular Disease.

[2] La Sala L., Pontiroli A.E. (2020). Prevention of Diabetes and Cardiovascular Disease in Obesity. Int. J. Mol. Sci..

[3] Antouri Z., Mezouaghi A., Djilali S., Zeb A., Khan I., Omer A.S.A. (2024). The impact of obesity on chronic diseases: Type 2 diabetes, heart disease, and high blood pressure. Appl. Math. Sci. Eng..

[4] Sanyaolu A., Okorie C., Qi X., Locke J., Rehman S. (2019). Childhood and Adolescent Obesity in the United States: A Public Health Concern. Glob. Pediatr. Health.

[5] Finkelstein E.A., Khavjou O.A., Thompson H., Trogdon J.G., Pan L., Sherry B., Dietz W. (2012). Obesity and Severe Obesity Forecasts Through 2030. Am. J. Prev. Med..

[6] Jaacks L.M., Vandevijvere S., Pan A., McGowan C.J., Wallace C., Imamura F., Mozaffarian D., Swinburn B., Ezzati M. (2019). The obesity transition: Stages of the global epidemic. Lancet Diabetes Endocrinol..

[7] Patel D. (2015). Pharmacotherapy for the management of obesity. Metabolism.

[8] Bray G.A., Frühbeck G., Ryan D.H., Wilding J.P. (2016). Management of obesity. Lancet.

[9] Kupnicka P., Król M., Żychowska J., Łagowski R., Prajwos E., Surówka A., Chlubek D. (2024). GLP-1 Receptor Agonists: A Promising Therapy for Modern Lifestyle Diseases with Unforeseen Challenges. Pharmaceuticals.

[10] Kang J.G., Park C.-Y. (2012). Anti-obesity drugs: A review about their effects and safety. Diabetes Metab. J..

[11] Cinkajzlová A., Mráz M., Haluzík M. (2022). Adipose tissue immune cells in obesity, type 2 diabetes mellitus and cardiovascular diseases. J. Endocrinol..

[12] Chylikova J., Dvorackova J., Tauber Z., Kamarad V. (2018). M1/M2 macrophage polarization in human obese adipose tissue. Biomed. Pap..

[13] Kawai T., Autieri M.V., Scalia R. (2021). Adipose tissue inflammation and metabolic dysfunction in obesity. Am. J. Physiol.-Cell Physiol..

[14] Kern L., Mittenbühler M.J., Vesting A.J., Ostermann A.L., Wunderlich C.M., Wunderlich F.T. (2019). Obesity-induced TNFα and IL-6 signaling: The missing link between obesity and inflammation—Driven liver and colorectal cancers. Cancers.

[15] Dai M., Yang X., Yu Y., Pan W. (2022). Helminth and host crosstalk: New insight into treatment of obesity and its associated metabolic syndromes. Front. Immunol..

[16] Su C.w., Chen C.-Y., Li Y., Long S.R., Massey W., Kumar D.V., Walker W.A., Shi H.N. (2018). Helminth infection protects against high fat diet-induced obesity via induction of alternatively activated macrophages. Sci. Rep..

[17] Kang S.A., Choi J.H., Baek K.-W., Lee D.I., Jeong M.-J., Yu H.S. (2021). Trichinella spiralis infection ameliorated diet-induced obesity model in mice. Int. J. Parasitol..

[18] Kang S.A., Yu H.S. (2024). Anti-obesity effects by parasitic nematode (*Trichinella spiralis*) total lysates. Front. Cell. Infect. Microbiol..

[19] Sumaiya K., Langford D., Natarajaseenivasan K., Shanmughapriya S. (2022). Macrophage migration inhibitory factor (MIF): A multifaceted cytokine regulated by genetic and physiological strategies. Pharmacol. Ther..

[20] Shi X., Leng L., Wang T., Wang W., Du X., Li J., McDonald C., Chen Z., Murphy J.W., Lolis E. (2006). CD44 is the signaling component of the macrophage migration inhibitory factor-CD74 receptor complex. Immunity.

[21] Kong Y.-Z., Chen Q., Lan H.-Y. (2022). Macrophage Migration Inhibitory Factor (MIF) as a Stress Molecule in Renal Inflammation. Int. J. Mol. Sci..

[22] Cho M.K., Lee C.H., Yu H.S. (2011). Amelioration of intestinal colitis by macrophage migration inhibitory factor isolated from intestinal parasites through Toll-like receptor 2. Parasite Immunol..

[23] Cho M.K., Park M.K., Kang S.A., Park S.K., Lyu J.H., Kim D.H., Park H.K., Yu H.S. (2015). TLR2-dependent amelioration of allergic airway inflammation by parasitic nematode type II MIF in mice. Parasite Immunol..

[24] Park S.K., Cho M.K., Park H.K., Lee K.H., Lee S.J., Choi S.H., Ock M.S., Jeong H.J., Lee M.H., Yu H.S. (2009). Macrophage migration inhibitory factor homologs of Anisakis simplex suppress Th2 response in allergic airway inflammation model via CD4+CD25+Foxp3+ T cell recruitment. J. Immunol..

[25] Sun H.-W., Swope M., Craig C., Bedarkar S., Bernhagen J., Bucala R., Lolis E. (1996). The subunit structure of human macrophage migration inhibitory factor: Evidence for a trimer. Protein Eng..

[26] Pandey H., Tang D.W.T., Wong S.H., Lal D. (2025). Helminths in alternative therapeutics of inflammatory bowel disease. Intest. Res..

[27] Maizels R.M., McSorley H.J. (2016). Regulation of the host immune system by helminth parasites. J. Allergy Clin. Immunol..

[28] van der Zande H.J.P., Zawistowska-Deniziak A., Guigas B. (2019). Immune Regulation of Metabolic Homeostasis by Helminths and Their Molecules. Trends Parasitol..

[29] Sikder S., Pierce D., Sarkar E.R., McHugh C., Quinlan K.G.R., Giacomin P., Loukas A. (2024). Regulation of host metabolic health by parasitic helminths. Trends Parasitol..

[30] de Luca C., Olefsky J.M. (2008). Inflammation and insulin resistance. FEBS Lett..

[31] Chen L., Chen R., Wang H., Liang F. (2015). Mechanisms Linking Inflammation to Insulin Resistance. Int. J. Endocrinol..

[32] Ziolkowska S., Binienda A., Jabłkowski M., Szemraj J., Czarny P. (2021). The Interplay between Insulin Resistance, Inflammation, Oxidative Stress, Base Excision Repair and Metabolic Syndrome in Nonalcoholic Fatty Liver Disease. Int. J. Mol. Sci..

[33] He A., Chen X., Tan M., Chen Y., Lu D., Zhang X., Dean J.M., Razani B., Lodhi I.J. (2020). Acetyl-CoA Derived from Hepatic Peroxisomal β-Oxidation Inhibits Autophagy and Promotes Steatosis via mTORC1 Activation. Mol. Cell.

[34] Zhong L., Huang L., Xue Q., Liu C., Xu K., Shen W., Deng L. (2019). Cell-specific elevation of Runx2 promotes hepatic infiltration of macrophages by upregulating MCP-1 in high-fat diet-induced mice NAFLD. J. Cell. Biochem..

[35] Nguyen T.T.P., Kim D.-Y., Lee Y.-G., Lee Y.-S., Truong X.T., Lee J.-H., Song D.-K., Kwon T.K., Park S.-H., Jung C.H. (2021). SREBP-1c impairs ULK1 sulfhydration-mediated autophagic flux to promote hepatic steatosis in high-fat-diet-fed mice. Mol. Cell.

[36] Smith K.R., Wang W., Miller M.R., Boucher M., Reynold J.E., Daurio N.A., Li D., Hirenallur-Shanthappa D., Ahn Y., Beebe D.A. (2024). GPAT1 Deficiency in Mice Modulates NASH Progression in a Model-Dependent Manner. Cell. Mol. Gastroenterol. Hepatol..

[37] Castro A.M., Macedo-de la Concha L.E., Pantoja-Meléndez C.A. (2017). Low-grade inflammation and its relation to obesity and chronic degenerative diseases. Rev. Méd. Hosp. Gen. Méx..

[38] Li M.C., He S.H. (2004). IL-10 and its related cytokines for treatment of inflammatory bowel disease. World J. Gastroenterol..

[39] Park H.J., Chung B.Y., Lee M.-K., Song Y., Lee S.S., Chu G.M., Kang S.-N., Song Y.M., Kim G.-S., Cho J.-H. (2012). Centipede grass exerts anti-adipogenic activity through inhibition of C/EBPβ, C/EBPα, and PPARγ expression and the AKT signaling pathway in 3T3-L1 adipocytes. BMC Complement. Altern. Med..

[40] Finucane O.M., Reynolds C.M., McGillicuddy F.C., Harford K.A., Morrison M., Baugh J., Roche H.M. (2014). Macrophage Migration Inhibitory Factor Deficiency Ameliorates High-Fat Diet Induced Insulin Resistance in Mice with Reduced Adipose Inflammation and Hepatic Steatosis. PLoS ONE.

[41] Baudry A., Yang Z.-Z., Hemmings B.A. (2006). PKBα is required for adipose differentiation of mouse embryonic fibroblasts. J. Cell Sci..

[42] Wang L., Zhang S., Cheng G., Mei C., Li S., Zhang W., Junjvlieke Z., Zan L. (2020). MiR-145 reduces the activity of PI3K/Akt and MAPK signaling pathways and inhibits adipogenesis in bovine preadipocytes. Genomics.

[43] Vermeire J.J., Cho Y., Lolis E., Bucala R., Cappello M. (2008). Orthologs of macrophage migration inhibitory factor from parasitic nematodes. Trends Parasitol..

[44] Karabowicz J., Długosz E., Bąska P., Wiśniewski M. (2022). Nematode Orthologs of Macrophage Migration Inhibitory Factor (MIF) as Modulators of the Host Immune Response and Potential Therapeutic Targets. Pathogens.

