# Supplementary material for: Recombinant Macrophage Migration Inhibitory Factor Derived from Trichinella spiralis Suppresses Obesity by Reducing Body Fat and Inflammation
Source: Int J Mol Sci. 2026 Jan 15;27(2):887. doi: 10.3390/ijms27020887 (PMC12841324; doi:10.3390/ijms27020887)
Supplement: Supplementary file 1 [file ijms-27-00887-s001.zip › Supplementary Figure S1.pdf]

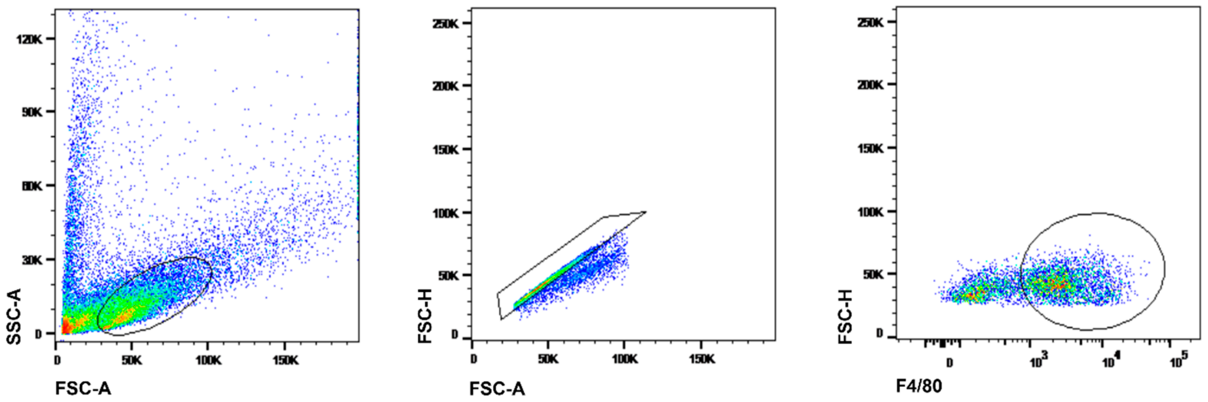

Supplementary Figure S1. Gating strategy for flow cytometric analysis of macrophage populations in eWAT. SVF cells isolated from eWAT were first gated based on forward scatter (FSC-A) and side scatter (SSC-A) to exclude debris. Doublets were excluded using FSC-A versus FSC-H gating. Macrophages were subsequently identified as F4/80⁺ cells. Within the F4/80⁺ population, M1 and M2 macrophages were defined based on the expression of CD11c and CD206, respectively. Different colors represent cell density, with warmer colors indicating higher event density. Circles indicate the gated cell populations used for subsequent analysis.
