# Supplementary material for: Recombinant Macrophage Migration Inhibitory Factor Derived from Trichinella spiralis Suppresses Obesity by Reducing Body Fat and Inflammation
Source: Int J Mol Sci. 2026 Jan 15;27(2):887. doi: 10.3390/ijms27020887 (PMC12841324; doi:10.3390/ijms27020887)
Supplement: Supplementary file 1 [file ijms-27-00887-s001.zip › Supplementary Table S1.pdf]

Supplementary Table S1. Flow cytometry conditions for analysis of adipose tissue macrophages

Category	Description
Tissue source	Epididymal white adipose tissue (eWAT)
Cell preparation	Digestion with 0.2% collagenase type I
Cell fraction analyzed	Stromal vascular fraction (SVF)
Primary macrophage marker	F4/80 (BL1/FITC, clone BM8)
M1 macrophage marker	CD11c^{+} (BL2/APC-Cy7, clone N418)
M2 macrophage marker	CD206^{+} (BL1/PE, clone C068C2)
Antibody source	eBioscience (San Diego, CA, USA)
Controls	Unstained controls and isotype controls
Flow cytometer	BD FACSCanto II (BD Biosciences)
Analysis software	FlowJo software (BD Biosciences)
